# A High Throughput *Barley Stripe Mosaic Virus* Vector for Virus Induced Gene Silencing in Monocots and Dicots

**DOI:** 10.1371/journal.pone.0026468

**Published:** 2011-10-21

**Authors:** Cheng Yuan, Cui Li, Lijie Yan, Andrew O. Jackson, Zhiyong Liu, Chenggui Han, Jialin Yu, Dawei Li

**Affiliations:** 1 State Key Laboratory of Agro-Biotechnology, China Agricultural University, Beijing, People's Republic of China; 2 Department of Plant and Microbial Biology, University of California, Berkeley, California, United States of America; Iwate University, Japan

## Abstract

*Barley stripe mosaic virus* (BSMV) is a single-stranded RNA virus with three genome components designated alpha, beta, and gamma. BSMV vectors have previously been shown to be efficient virus induced gene silencing (VIGS) vehicles in barley and wheat and have provided important information about host genes functioning during pathogenesis as well as various aspects of genes functioning in development. To permit more effective use of BSMV VIGS for functional genomics experiments, we have developed an *Agrobacterium* delivery system for BSMV and have coupled this with a ligation independent cloning (LIC) strategy to mediate efficient cloning of host genes. Infiltrated *Nicotiana benthamiana* leaves provided excellent sources of virus for secondary BSMV infections and VIGS in cereals. The Agro/LIC BSMV VIGS vectors were able to function in high efficiency down regulation of phytoene desaturase (*PDS*), magnesium chelatase subunit H (*ChlH*), and plastid transketolase (*TK*) gene silencing in *N. benthamiana* and in the monocots, wheat, barley, and the model grass, *Brachypodium distachyon*. Suppression of an *Arabidopsis* orthologue cloned from wheat (*TaPMR5*) also interfered with wheat powdery mildew (*Blumeria graminis* f. sp. *tritici*) infections in a manner similar to that of the *A. thaliana* PMR5 loss-of-function allele. These results imply that the *PMR5* gene has maintained similar functions across monocot and dicot families. Our BSMV VIGS system provides substantial advantages in expense, cloning efficiency, ease of manipulation and ability to apply VIGS for high throughput genomics studies.

## Introduction

Virus-induced gene silencing (VIGS) is a particularly useful tool for plant functional genomics because it permits knockdown of genes-of-interest and observation of elicited phenotypes within 3 to 4 weeks. VIGS avoids production of knockout mutants or stable RNA interference (RNAi) and can also be performed on species that are difficult to transform [1], [2], [3], [4]. The VIGS principle is based on antiviral responses that target RNAs for degradation and is triggered by accumulation of double-stranded RNAs (dsRNA) appearing in the infection cycle [5], [6]. During the early, or “shock”, phase of infection intense symptoms develop and high levels of virus accumulate. As host defense mechanisms are activated, infections usually transit into a protracted chronic phase characterized by modulated symptoms and low virus abundance. By inserting sequence fragments derived from “genes-of-interest” into VIGS vectors, the corresponding mRNAs are selectively degraded during virus infection to result in transient silencing of the targeted gene.

The first VIGS vectors were derived from *Tobacco mosaic virus* (TMV) [7], *Potato virus X* (PVX) [8], [9] and *Tobacco rattle virus* (TRV) [10], [11], and these vectors were initially used for *Nicotiana benthamiana* and tomato (*Solanum lycopersicum*) gene silencing. Recently, the plants in which VIGS has been employed has increased substantially, and more than 30 viruses have been shown to have potential as VIGS vectors. Examples include *Apple latent spherical virus* (ALSV) [12], [13], *Bean pod mottle virus* (BPMV) [14], *Brome mosaic virus* (BMV) [15], [16], *Pea early browning virus* (PEBV) [17], *Rice tungro bacilliform virus* [18], *Tomato bushy stunt virus* [19], *Turnip yellow mosaic virus* (TYMV) [20], and some Geminiviruses and their satellite DNAs [21], [22].

*Barley stripe mosaic virus* (BSMV) vectors suitable for cereal VIGS have also been described [23], [24], [25], [26], [27], [28], [29]. BSMV is a positive-sense RNA virus with a broad experimental host range and is the type member of the *Hordeivirus* genus [30], [31]. The tripartite genome consists of RNAs α, β and γ, and each of the genomic (g) RNAs has a methylated 5′ cap and a 3′ polyadenylate sequence followed by a tyrosine accepting tRNA-like structure. RNAα of the BSMV ND18 strain encodes the methyltransferase/helicase subunit of the RNA-dependent RNA polymerase (RdRp). RNAβ specifies the coat protein (CP) and three major triple gene block (TGB) proteins (TGB1, TGB2 and TGB3) that are essential for cell-to-cell movement of the virus [30], [31]. RNAγ encodes the polymerase (GDD) subunit of the RdRp and the γb protein, which has significant roles in viral pathogenesis, long distance movement and suppression of host RNA silencing defenses [30], [31], [32], [33].

BSMV was first modified as a VIGS vector [25] for use in barley (*Hordeum vulgare*), and subsequently was used to down regulate expression of wheat (*Triticum aestivum*) genes [28]. Applications of BSMV-based VIGS include functional genomics research in wheat [28], [34], [35], [36], barley [25], [26], [27], [29], [37], *Dasyprum villosum* (*Haynaldia villosa*) [38], [39], a distantly related wild wheat relative, *Brachypodium distachyon* [40], [41], a model organism for cereals, and the culinary ginger (*Zingiber officinale*), a tropical monocot [42]. In these studies, fragments from genes-of-interest were initially inserted into RNAγ either downstream or upstream of the γb gene and shown to elicit visual phenotypes, or to function in morphogenesis or disease responses [2]. For example, BSMV VIGS has been used to disrupt several wheat resistance pathways, including *Lr1*-, *Lr10*- and *Lr21*-mediated leaf rust resistance [28], [34], [36], [43], stripe rust resistance [35], functional alleles in the *Pm3* powdery mildew resistance locus [44], and *Stpk-V*, a key member of the *Pm21* powdery mildew resistance gene complex [39]. Several barley studies have focused on powdery mildew *Mla13*-mediated resistance [29], stem rust *Rpg5* R-gene regulation [45], the roles of a susceptibility factor, *HvBI-1*, that modulates cell wall-associated defenses [46], and nonhost resistance to *Cochliobolus carbonum* [47]. More recently, the Wise laboratory has developed a BSMV-based VIGS plasmid to evaluate resistance genes involved in barley-powdery mildew interactions [26], [27]. In a novel approach that has been called “host-induced gene silencing” (HIGS), the possibility of down regulating pathogen genes with BSMV VIGS has been shown to silence a wheat powdery mildew (*Blumeria graminis* f. sp. *tritici*) or rust fungi (*Puccinia striiformis* f. sp. *tritici*) gene and to result in effective interference with infection of wheat [48], [49]. BSMV VIGS also has potential for determining aphid defense gene functions in wheat [50]. In this case, a *WKRY53* transcription factor and an inducible phenylalanine ammonia-lyase (*PAL*) were shown to have key roles in resistance responses to aphid (*Diuraphis noxia*) infestations. In addition BSMV-VIGS has been adapted for studies of morphogenesis and development in crop plants [51], [52], [53], [54].

The first generation BSMV VIGS systems under the control of the T7 promoter [2] were time consuming and expensive because capped *in vitro* transcripts from the α, β and γ cDNA clones are required for plant inoculation. The latter problem has been circumvented in the Wise lab [26], [27] by engineering plasmids containing each of the cDNAs, and incorporating a double *Cauliflower mosaic virus* (CaMV) 35S promoter immediately upstream of the cDNAs and a *Hepatitis delta virus* (HDV) ribozyme immediately downstream of the cDNAs. Biolistic introduction of these plasmids into barley leaves resulted in replication of BSMV VIGS derivatives containing candidate genes predicted to affect powdery mildew resistance, and sap from the systemically infected leaves was suitable for secondary inoculations to other cereals. However, a limitation of the Wise method is the potential instability of gene inserts that can occur during systemic invasion of the primary inoculated plants [55], [56], [57]. Another limitation of both strategies is that cloning multiple genes-of-interest can be time-consuming and inefficient when high throughput operations are required.

To permit more effective use of BSMV VIGS for functional genomics experiments, we have developed an approach with *Agrobacterium tumefaciens* strains harboring the BSMV α, β, and γ cDNAs in Ti plasmids for initiation of BSMV infections upon infiltration of *N. benthamiana* leaves. The *Agrobacterium* mediated BSMV VIGS vectors were engineered by inserting BSMV cDNAs between the double 35S promoter and a ribozyme sequence (Rz) from *Tobacco ringspot virus* (TRSV) satellite RNA [58]. In addition, we have inserted a ligation independent cloning (LIC) site similar to that used for TRV VIGS [59] into BSMV to facilitate efficient cloning of desired gene fragments. These two modifications permit rapid VIGS analyses of large numbers *N. benthamiana* plants, as we have demonstrated with phytoene desaturase (*PDS*) and plastid transketolase (*TK*) genes [60]. Infiltrated *N. benthamiana* leaves also accumulated high levels of BSMV and provided excellent sources for secondary infections to elicit VIGS in wheat, barley, and the model grass, *B. distachyon* [61], [62]. We also have used BSMV Agro/LIC VIGS to clone and analyze a wheat powdery mildew resistance 5 gene (*TaPMR5*) fragment whose *Arabidopsis thaliana* PMR5 (*AtPMR5*) loss-of-function allele elicits resistance to two powdery mildew fungi, *Golovinomyces* (formerly *Erysiphe*) *cichoracearum* and *G*. *orontii*, [63]. Transient down regulation of PMR5 compromises the ability of wheat powdery mildew (*B. graminis* f. sp. *tritici*) to establish infections, and illustrates the cross kingdom requirements of PMR5 for powdery mildew invasion of monocot and dicot hosts.

## Results

### *Agrobacterium* mediated BSMV infectivity of *N. benthamiana*, wheat, barley and *B. distachyon*

**Figure 1:**
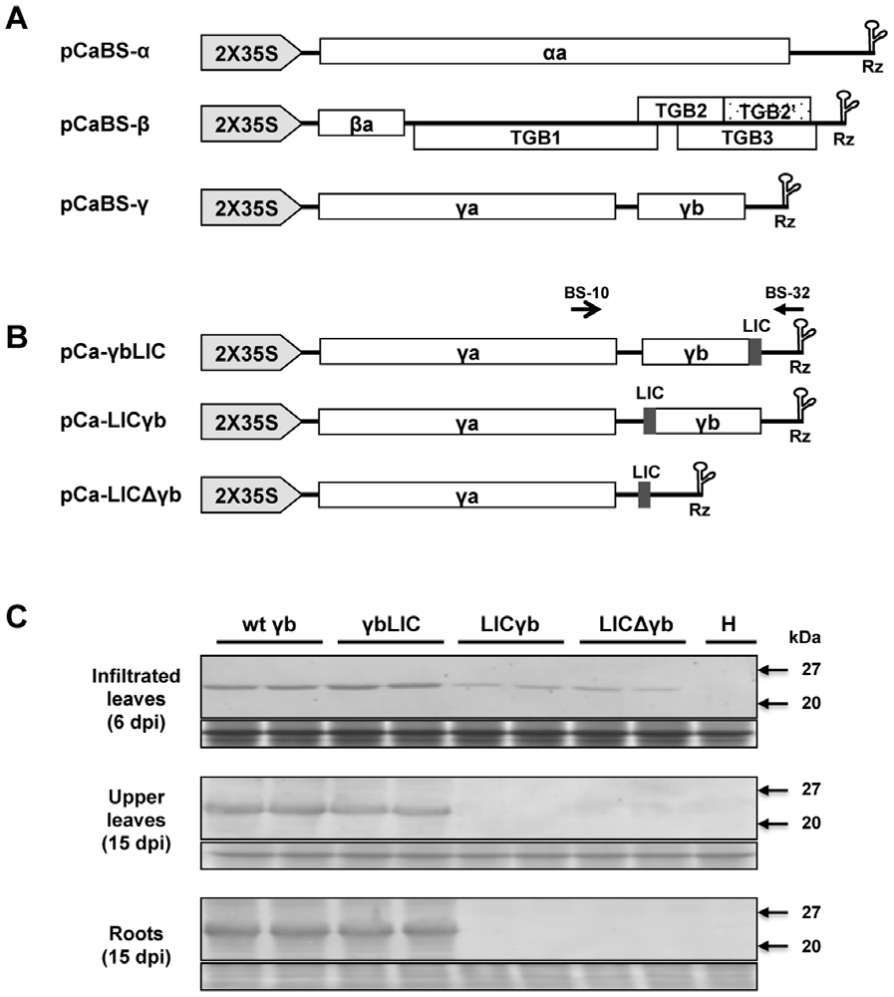
Construction of *Agrobacterium* mediated BSMV VIGS vectors with LIC cloning sites and comparisons of BSMV accumulation in *N. benthamiana* tissues infected with wild type BSMV and its three LIC derivatives. (**A**) Schematic representation of pCaBS-α, pCaBS-β and pCaBS-γ. *Agrobacterium* VIGS plasmids were engineered by inserting the respective BSMV α, β and γ cDNAs between the double CaMV 35S promoter and a ribozyme sequence (Rz) derived from TRSV satellite RNA. (**B**) The organization of three LIC derivatives derived from pCaBS-γ. The LIC site was inserted into pCaBS-γ at three positions downstream of the γa gene. The pCa-γbLIC plasmid was designed to express a full-length γb protein containing two C-terminal amino acid (Glu and Val) extensions preceding the VIGS target sequence. For pCa-LICγb, the LIC site was engineered to replace the AUG start codon of the γb gene and eliminate γb protein expression, and in the case of pCa-LICΔγb, the LIC site was substituted for the γb gene. The primer pairs BS-10 and BS-32 (Table S1), whose positions are indicated above pCa-γbLIC, were used for PCR amplifications to assess the stability of fragments inserted into the pCa-γbLIC vectors. (**C**) Western blot analysis of BSMV infections after agroinfiltration of *N. benthamiana* leaves. Analyses of infiltrated leaves are shown at 6 dpi to illustrate virus accumulation before spread to the vascular system. Upper uninfiltrated leaves and roots were assessed at 15 dpi to compare the accumulation of BSMV pCaBS-γ and its three LIC derivatives. Protein was isolated from two plants agroinfiltrated with pCaBS-α and pCaBS-β mixtures containing pCaBS-γ (wt-γb), pCa-γbLIC (γbLIC), pCa-LICγb (LICγb) or pCa-LICΔγb (LICΔγb). BSMV coat protein was detected with BSMV antisera and healthy *N. benthamiana* leaves (H) were used as negative controls. The bottom panel shows a loading control consisting of Coomassie Blue stained Rubisco, the major protein associated with tobacco leaf extracts.

The three BSMV ND18 cDNAs [64] were cloned into the pCass4-Rz T-DNA vector under the control of a double 35S promoter [58] to permit precise *in vivo* transcription at the 5′ terminus of the gRNAs. The cDNA α clone was inserted into the *Stu*I site of pCass4-Rz, and the cDNAβ and γ clones were integrated between *Stu*I and *Bam*HI sites to produce pCaBS-α, pCaBS-β and pCaBS-γ (Fig. 1A). The TRSV satellite RNA ribozyme mediated cis-cleavages extending 33, 17 and 17 nts non-viral sequences beyond the 3′ ends of RNAα, β and γ respectively.

**Figure 2:**
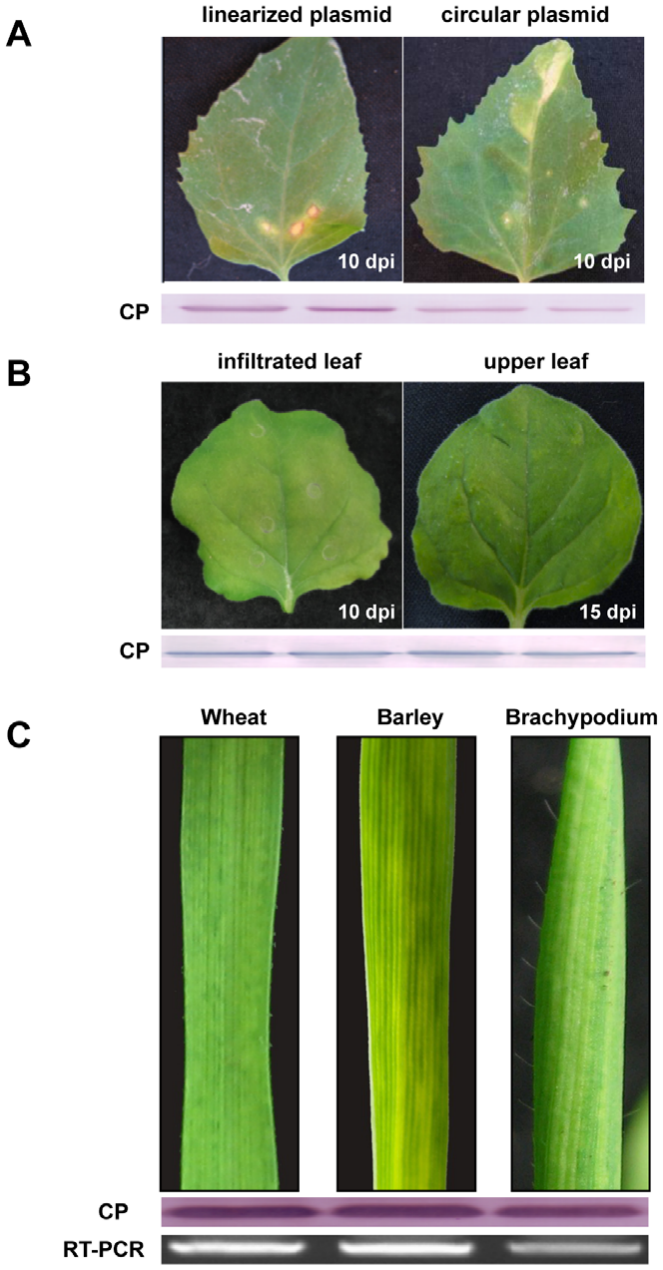
*Agrobacterium* mediated BSMV infection of *N. benthamiana*, wheat, barley and *B. distachyon*. (**A**) Appearance of large necrotic local lesions on leaves of *C. amaranticolor* at 10 days after mechanical inoculation with either circular (right) or linearized (left) plasmids. CP shows western blots of BSMV CP isolated from two separate leaves. (**B**) *N. benthamiana* leaves infiltrated with equal concentrations of *Agrobacterium* mixtures harboring pCaBS-α, pCaBS-β, and pCaBS-γ. Infiltrated leaves at 10 dpi (left) and upper uninfiltrated leaves developing mild mottling symptoms at 15 dpi (right). CP is as designated panel A. (**C**) Typical BSMV symptoms on systemically infected leaves of wheat, barley and *B. distachyon* emerging at 14 dpi after inoculation of plants with infected *N. benthamiana* leaf sap. The presence of BSMV RNA and CP was confirmed by RT-PCR and Western blots.

To confirm the infectivity of pCaBS-α, pCaBS-β and pCaBS-γ, the plasmids were mixed and rub-inoculated on *Chenopodium amaranticolor* leaves. Large necrotic lesions similar to those resulting from BSMV RNAs or purified BSMV inoculum appeared at 7 to 10 days post-inoculation (dpi) only on leaves abraded with either circular or linearized plasmids (Fig. 2A). After infiltration with equal concentrations of *A. tumefaciens* EHA105 mixtures harboring pCaBS-α, pCaBS-β and pCaBS-γ, the upper uninfiltrated *N. benthamiana* leaves developed mild mottling symptoms at 7 to 10 dpi. Western blotting revealed high levels of coat protein, and PCR analyses verified the presence of the gRNAs (data not shown), indicating systemic movement from the infiltrated leaves (Fig. 2B).

Different *Agrobacterium* strains (EHA105, EHA101 and GV3101, harboring pCaBS-α, pCaBS-β and pCaBS-γ) were used to infiltrate barley and wheat, but none of these resulted in BSMV infections. Other inoculation methods were also tested without success, including mechanical inoculation with circular or linearized plasmids, and vacuum infiltration of germinated seeds or seedlings with the *Agrobacterium* derivatives. Consequently, infiltrated *N. benthamiana* leaf sap was used to inoculate wheat and barley. More than 80% of the uninoculated emerging leaves of both cereals consistently developed typical mosaic symptoms at 6 to 7 dpi, and the presence of BSMV RNA and CP was confirmed by RT-PCR and Western blots (Fig. 2C). The inbred *B. distachyon* line Bd21-3 also consistently developed a mild systemic phenotype (Fig. 2C), similar to that shown in more limited earlier studies [41] Moreover, in all cases BSMV symptoms elicited by the ND18 strain used in these studies (Fig. 2C) were clearly distinct from the photobleaching effects and other visible VIGS phenotypes described in the sections below. Taken together, our results demonstrate that agroinfiltrated leaves of *N. benthamiana* are excellent sources of BSMV VIGS inoculum for high throughput dissection of wheat, barley and *B. distachyon* genes involved in disease development and pathogenesis.

### A LIC strategy for high-throughput VIGS

**Figure 3:**
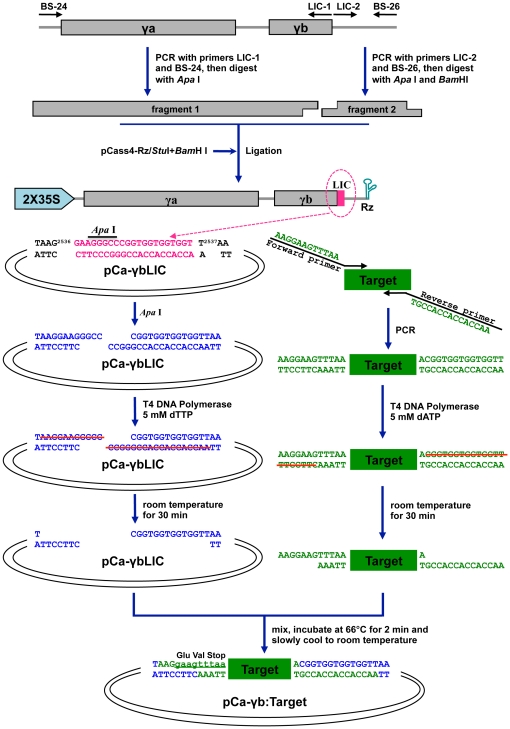
Schematic representation of use of the pCa-γbLIC vector for target gene fragment cloning. Individual steps in LIC cloning and insertion of target fragments to be used for VIGS assessment are illustrated.

One limitation of *in vitro* transcription and DNA based BSMV VIGS vectors [25], [27] is the relatively inefficient cloning of target genes needed for efficient high throughput VIGS operations. To circumvent this limitation, a LIC site (5′-GAAGGGCCCGGTGGTGGTGGT-3′) with an *Apa*I site (underlined) was inserted into pCaBS-γ at three positions downstream of the γa (polymerase subunit) gene. The plasmid pCa-γbLIC was engineered by integrating the LIC site immediately upstream of the stop codon of the γb coding region, to provide a functional γb protein with an extension of two C-terminal residues (Glu and Val) after inserting target fragments (Fig. 1B and Fig. 3). For pCa-LICγb, the LIC site was designed to replace the γb AUG start codon and eliminate γb protein expression, and the LIC site was engineered as a replacement for γb to generate pCa-LICΔγb (Fig. 1B).

Western blot analysis of *N. benthamiana* leaves at 6 days after infiltration with *Agrobacterium* mixtures containing pCaBS-α, pCaBS-β and one of the three pCaBS-γ LIC derivatives revealed the presence of BSMV coat protein in all three combinations. However, leaves infiltrated with pCa-LICγb and pCa-LICΔγb mixtures accumulated reduced levels of coat protein (37.5% and 46.2% of pCaBS-γ). Moreover, at 15 dpi only pCa-γbLIC accumulated to the same levels as wild-type pCaBS-γ in the upper uninfiltrated leaves, and only wt pCaBS-γ and pCa-γbLIC were detected in the upper uninfiltrated leaves and the roots (Fig. 1C). These results indicated that although the three BSMV derivatives can replicate in infiltrated tissue, γb expression is important for efficient vascular movement in *N. benthamiana*, as shown previously [65], [66]. The efficiency of LIC cloning into the pCa-γbLIC site was also confirmed by recovery of *PDS* gene fragments from different plant species. In these experiments, 43 of 44 clones had inserts corresponding in size to the targeted inserts (data not shown). Therefore, pCa-γbLIC was chosen for all subsequent BSMV VIGS experiments.

### Agrobacterium mediated BSMV VIGS in *N. benthamiana*

*N. benthamiana* is the most widely used experimental dicot host for VIGS because of its susceptibility to a large number of plant viruses and the rapidity with which visible phenotypes appear [67]. To assess silencing by *Agrobacterium* mediated BSMV VIGS, a 370 bp *PDS* (*NbPDS*) fragment and a 400 bp *TK* (*NbTK*) fragment from *N. benthamiana* were cloned into pCa-γbLIC in the sense orientations to generate pCa-γb:*NbPDS*_370_ and pCa-γb:*NbTK*_400_, respectively. Then, the four-leaf stage of *N. benthamiana* was infiltrated with *Agrobacterium* mixtures containing pCaBS-α, pCaBS-β and pCa-γb:*NbPDS*_370_ or pCa-γb:*NbTK*_400_.

**Figure 4:**
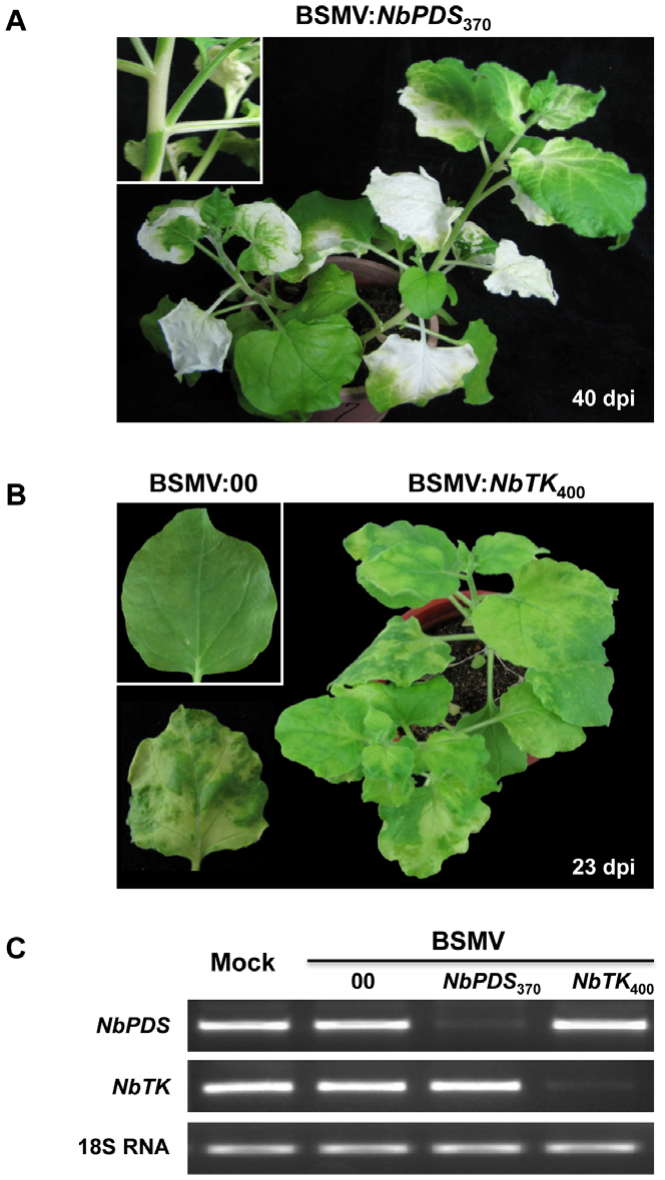
Silencing of endogenous phytoene desaturase (*PDS*) and plastid transketolase (*TK*) genes in *N. benthamiana* by *Agrobacterium* mediated BSMV VIGS. The four-leaf stage of *N. benthamiana* was infiltrated with an *Agrobacterium* mixture containing pCaBS-α, pCaBS-β and pCa-γb:*NbPDS*_370_ (BSMV:*NbPDS*_370_) or pCa-γb:*NbTK*_400_ (BSMV:*NbTK*_400_). (**A**) Large areas of photobleaching occurring in leaves infected with BSMV:*NbPDS*_370_ 40 dpi, and an intense white photobleaching often appearing in stems and petioles (see magnified inset panel). (**B**) Chlorotic sectors with a distinctive carotenoid coloration interspersed with pale green regions in upper uninfiltrated leaves of plants infected at 23 dpi with BSMV:*NbTK*_400_. A leaf of a plant infiltrated with the empty vector (BSMV:00) is shown in the inset panel. (**C**) Relative transcript levels of *NbPDS* and *NbTK* in leaves infected with the BSMV:00, BSMV:*NbPDS*_370_ or BSMV:*NbTK*_400_. RNA extracted from the leaves was subjected to semi-quantitative RT-PCR amplification (27 cycles) with the gene-specific oligonucleotide primers shown in Table S1. Amplified tobacco 18S rRNA served as an internal control.

Leaves infiltrated with pCaBS-α, pCaBS-β and pCa-γb:*NbPDS*_370_ to elicit *PDS* silencing developed a mottled photobleaching phenotype in the 5th or 6th leaves at 9 to 10 dpi, and about 5 days later (15 dpi) larger areas of more uniform white *PDS* silencing were observed at the 6 to 8 leaf stage (data not shown). *PDS* silencing was most pronounced at 30 to 45 dpi when large areas of white photobleaching were evident in most leaves and, in many cases, on stems and petioles (Fig. 4A). Leaf and stem silencing persisted until the leaves began to senesce, but silencing in younger emerging leaves began to gradually dissipate as green sectors at the tips and margins of the leaves became increasingly evident.

The *TK* gene, used for *N. benthamiana* VIGS, encodes an enzyme required for photosynthesis and phenylpropanoid metabolism [60]. When pCa-γb:*NbTK*_400_ was co-infiltrated with pCaBS-α, pCaBS-β, an obvious chlorosis became evident at 9 to 10 dpi on the 5th or 6th emerging leaves (data not shown). The chlorosis gradually transitioned into a more generalized silencing phenotype and by 23 dpi all upper uninfiltrated leaves displayed yellowing chlorotic sectors with a distinctive carotenoid coloration interspersed with pale green regions (Fig. 4B) that appear to be similar to the *TK* knockdown phenotype noted in previous studies [68].

Overall, silencing was efficient with the *NbPDS* and *NbTK* genes and, in most experiments, distinctive symptoms appeared in >80% of the infiltrated plants grown under our growth chamber conditions. The lower transcript abundances of the *NbPDS* and *NbTK* genes in *N. benthamiana* infected with BSMV:*NbPDS*_370_ or BSMV:*NbTK*_400_ respectively were confirmed by semi-quantitative RT-PCR and by use of a tobacco 18S rRNA as an internal control (Fig. 4C). To the best of our knowledge, this is the first report of BSMV-based VIGS in *N. benthamiana*, and it is notable that the high efficiency and intensity of the silencing phenotypes are comparable to those triggered by other VIGS vectors [4]. Hence, our results demonstrate that BSMV VIGS is a viable choice for use in conjunction with *N. benthamiana* genomics studies.

### BSMV accumulation and insert stability in infiltrated *N. benthamiana* leaves

**Figure 5:**
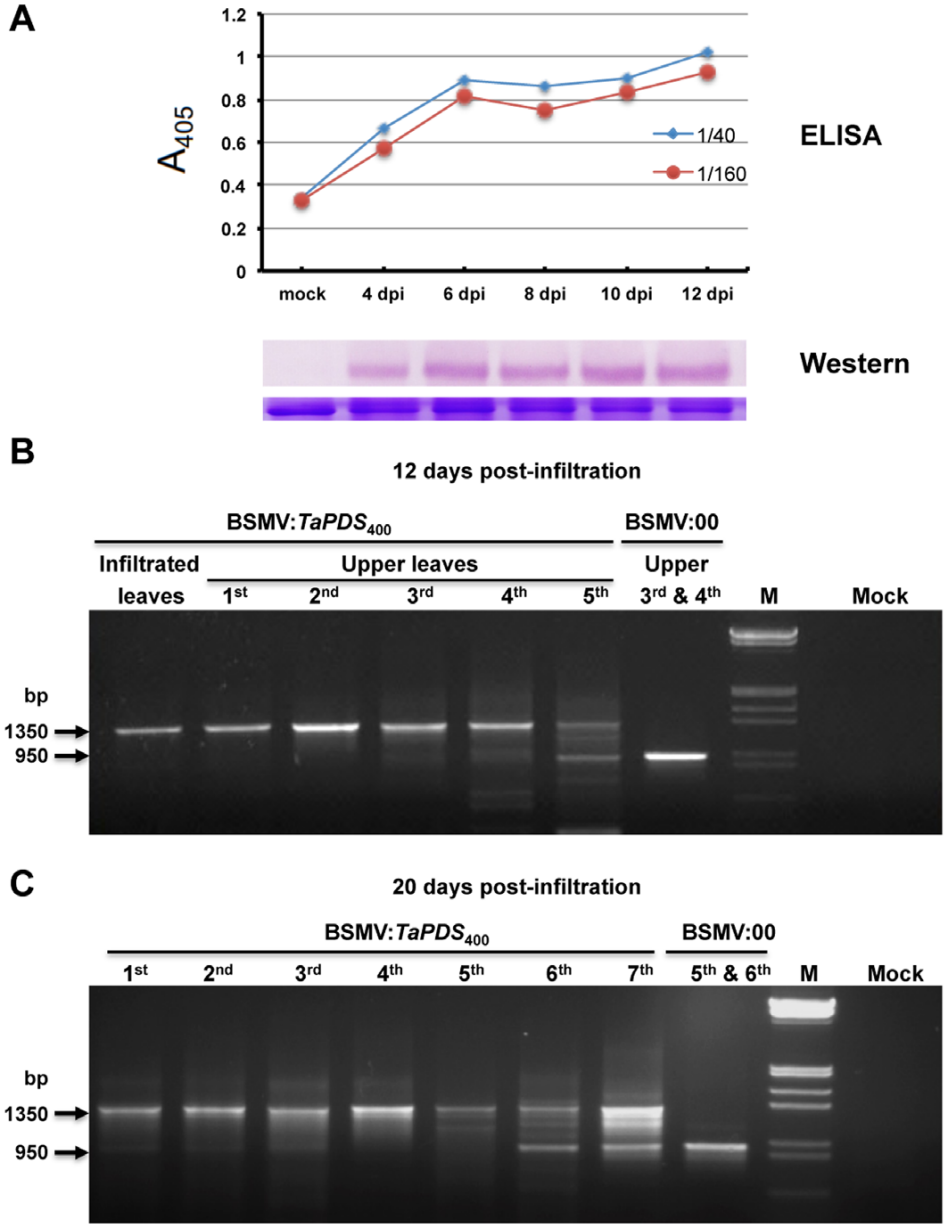
Stability of inserted fragments in *N. benthamiana* leaves after infiltration with BSMV:*TaPDS*_400_. (**A**) Time course of BSMV accumulation in infiltrated *N. benthamiana* leaves. Infiltrated leaves were harvested at 4, 6, 8, 10 and 12 days post infiltration. The two curves in the graph represent ELISA analyses of 1∶40 and 1∶160 sample dilutions. The western blot provides an independent assessment of BSMV coat protein accumulation in the infiltrated leaves. The lower band shows a Rubisco loading control. (**B**) Leaves infiltrated with BSMV:*TaPDS*_400_, and the first 5 leaves above the infiltrated leaves were harvested at 12 dpi, and amplified by RT-PCR with the primer pair BS-10/BS-32 (Fig. 1B; Table S1) to assess insert stability during systemic movement. The third and fourth leaves above the leaves infiltrated with the pCa-γbLIC (BSMV:00) were amplified as controls. Note: A distinct shadow progressively appears below the main 1350 bp band in systemically infected leaves 2 to 4, and an obvious smaller band of ∼950 bp corresponding to the empty LIC site appears in the 5th upper leaves. (**C**) The first 7 leaves above the infiltrated leaves infiltrated with BSMV:*TaPDS*_400_, and the upper 5th and 6th leaves infiltrated with BSMV:00 were harvested for RT-PCR at 20 dpi. In this case, the deletion products corresponding in size to the BSMV:00 fragment were more evident in the 6th and 7th leaves and represent a substantial proportion of the PCR population.

Because a high percentage of plants consistently developed gene silencing phenotypes after agroinfiltration of *N. benthamiana*, we wished to determine the accumulation of BSMV to provide a basis for efficient transfer of BSMV vectors suitable for VIGS elicitation in secondary plants. When the first four to eight leaves above the *N. benthamiana* cotyledons were infiltrated, the levels of BSMV coat protein increased for the first 6 days and then leveled off and remained relatively constant for the next six days (Fig. 5A). Moreover, several grams of tissue were recovered from the infiltrated leaves and extracts from this tissue were able to provide >80% infections of cereals used for VIGS tests (data not shown). These results demonstrate that *N. benthamiana* amplification of BSMV from primary infiltrated leaves provides an inexpensive inoculum source for VIGS trials of a large number of plants.

To compare the stability of inserts in infiltrated and systemically infected leaves at different times after infection, *N. benthamiana* leaves were infiltrated with pCa-γb:*TaPDS*_400_. Then at 12 dpi, the next four fully expanded leaves above the infiltrated leaf, and the fifth emerging leaf, which was still expanding, were harvested. The primer pair BS-10/BS-32 flanking the LIC site (Fig. 1B; All primers used in this work are listed in Table S1) was used for RT-PCR of RNA extracted from each leaf. Although agarose gels revealed that the inserted sequence is relatively stable in the infiltrated leaves, a distinct shadow below the major 1350 bp band became progressively more intense in the upper systemically infected leaves 1 to 4 (Fig. 5B). In addition, an obvious smaller band of ∼950 bp, whose size is similar to the RT-PCR products of the empty pCa-γbLIC vector appeared in the 5th emerging smaller leaves. Moreover, degradation products were more pronounced in upper systemically infected leaves at 20 dpi (Fig. 5C). At this time, the infiltrated leaves were becoming necrotic, so we were unable to isolate intact RNA at later stages. Taken together, these results suggest that infiltrated leaves harvested within 12 days after BSMV infection contain a high abundance of intact inserts with negligible amounts of degradation products, and should be the most suitable source for secondary inoculations for VIGS tests. This is particularly important for VIGS analyses with larger inserts because infiltrated leaves maximizes foreign sequence stability and minimizes loss of inserts occurring during long distance BSMV movement [55], [56], [57].

### *Agrobacterium* mediated BSMV VIGS in cereals

**Figure 6:**
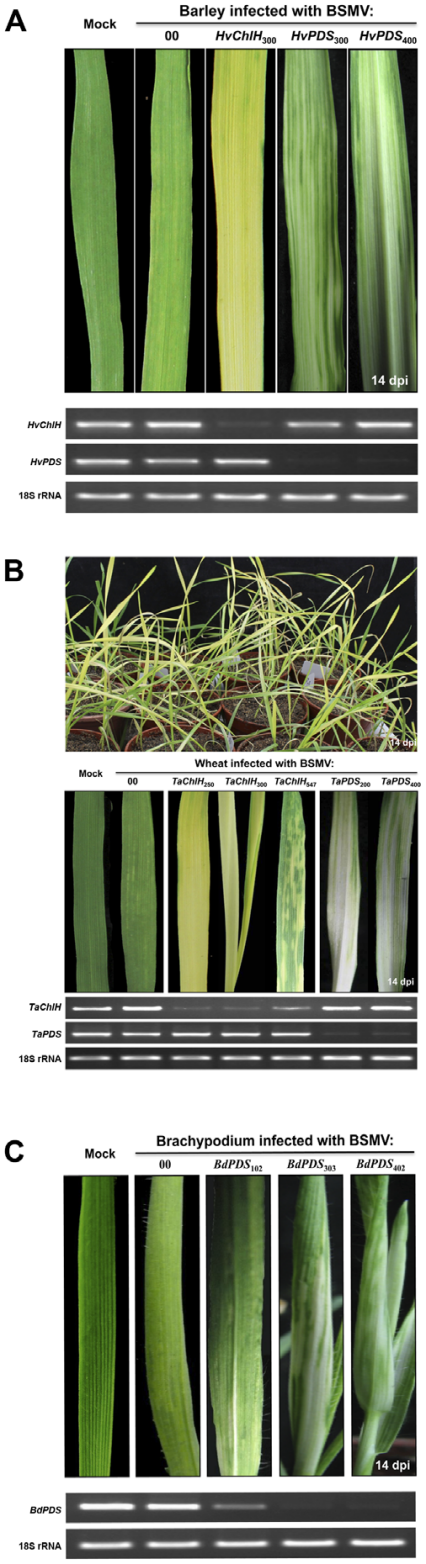
*PDS* and magnesium chelatase subunit H (*ChlH*) gene silencing phenotypes in barley, wheat and *B. distachyon*. (**A**) Barley showing phenotypes typical of suppression of *ChlH* and *PDS* by BSMV:*HvChlH* or BSMV:*HvPDS* inserts of the indicated lengths. (**B**) **Upper panel**: Wheat inoculated with BSMV:*TaChlH_250_* showing more than 90% of inoculated plants developing the chlorotic phenotype associated with suppression of the *ChlH* gene. **Middle panel**: *PDS* and *ChlH* gene silencing phenotypes on wheat leaves infected with BSMV:*TaChlH* and BSMV:*PDS* derivatives with different length inserts. **Bottom panel**: PCR amplification of transcripts from leaves shown in the middle panel. (**C**) *B. distachyon* leaves showing effects of *PDS* suppression after infection with BSMV:*BdPDS* inserts. In these experiments, plants were inoculated at the two-leaf stage with infected *N. benthamiana* sap harboring BSMV derivatives targeting *PDS* and *ChlH* cognate genes from each species. Leaf photographs were taken at 14 dpi, and fragment lengths from each source are indicated as subscripts above each leaf. Relative transcript levels of *PDS* and *ChlH genes* in the leaves infected with the different BSMV derivatives are shown under the leaf photographs. RNA extracted from the leaves was subjected to semi-quantitative RT-PCR amplification (28 cycles for wheat and barley, 31 cycles for *B. distachyon*) with the gene-specific oligonucleotide primers shown in Table S1. Amplified species-specific 18S rRNA served as internal controls for each species.

Although we were unable to agroinfect cereals directly, *N. benthamiana* infiltrated leaves provided an excellent source for secondary inoculations to wheat, barley, and the model grass, *B. distachyon* (Fig. 3; Table S2). Using LIC cloning techniques, a series of *PDS* or magnesium chelatase subunit H (*ChlH*) target gene fragments of different lengths from each species to be tested were cloned into the LIC site of pCa-γbLIC (Fig. 3). When the first two emerging leaves of wheat, barley, and *B. distachyon* were inoculated with infected *N. benthamiana* sap, silencing phenotypes appeared 10 to 14 dpi on upper leaves emerging after infection that corresponded to the cognate *PDS* or *ChlH* sequences were used for silencing (Fig. 6; Fig. S1; Table S2). Leaves of all three cereal species targeted for down regulation of *PDS* developed a longitudinal white streaks that appeared to be devoid of carotenoid pigment. These photobleached segments occupied various portions of the parallel venation and were interspersed within green regions of the leaf blades. In the case of the *ChlH* phenotype, a more intense yellowing sometimes covered large areas of the leaf, but the yellowing was often interspersed with green sections. In contrast, BSMV or BSMV:00 infected leaves developed typical mosaic symptoms consisting of mild light yellow chlorotic streaks (Fig. 6 and Fig. S1). In all three species, the virus symptoms could be easily distinguished visually from the *PDS* or *ChlH* phenotypic effects. In most cases, a high proportion of the plants (>80%) developed the silencing phenotype as illustrated in Fig. 6B, which shows several pots of wheat at 14 dpi, in which >90% of the plants exhibit the chlorotic phenotype associated with suppression of *TaChlH* transcripts.

To determine the effects of fragment lengths on silencing efficiency, we evaluated phenotypes induced by 200 and 400 bp *TaPDS* fragments, and 250, 300 and 547 bp *TaChlH* fragments in wheat, a 300 bp *HvChlH* fragment, 300 and 400 bp *HvPDS* fragments in barley, and 102, 303 and 402 bp *BdPDS* fragments in brachypodium (Fig. 6). The 300 and 400 bp *HvPDS* fragments appeared to elicit similar levels of photobleaching in barley leaves (Fig. 6A). The extent of silencing in wheat appeared to be substantially more efficient with the 250 bp and 300 bp *TaChlH* inserts than the 547 bp insert. In these cases, the two smaller inserts consistently induced more uniform bright yellowing phenotypes than the 547 bp insert, which often elicited a mottled phenotype on emerging leaves. The results of semi-quantitative RT-PCR also show that the *TaChlH* transcript abundance was higher in BSMV:*TaChlH*_547_ infected wheat than BSMV:*TaChlH*_250_ or BSMV:*TaChlH*_300_ infections, and that *TaPDS* RNA levels were reduced to similar levels in BSMV:*TaPDS*_200_ or BSMV:*TaPDS*_400_ infected leaves (Fig. 6B). In another comparison, the 102 bp *BdPDS* fragment was less effective than the 303 bp or 402 bp fragments in induction of photobleaching in *B. distachyon*, and these photo bleaching phenotypes were consistent with the *BdPDS* transcript abundance assayed by semi-quantitative RT-PCR (Fig. 6C). These results indicate that sequences ranging from 200 to 400 bp provide effective silencing with BSMV VIGS, and that the gene silencing is specific for the cognate gene fragments used to induce silencing and for the anticipated silencing phenotype [2], [3], [24].

### Functional analysis of a gene required for powdery mildew invasion

The potential of *Agrobacterium* mediated BSMV VIGS for analyses of genes involved in disease development was assessed by investigating a orthologue of the Arabidopsis *PMR5* gene, which is known to interfere with powdery mildew (*Golovinomyces cichoracearum* and *G. cichoracearum*) colonization of *A. thaliana* [63]. To determine the possible presence of a wheat orthologue of *PMR5*, we carried out a BLAST search with the *A. thaliana PMR5* gene sequence and found a 337 bp region in common wheat with a 60.5% nucleotide sequence identity. Because this 337 bp region has 100% identity in the wheat Chinese Spring and Xuezao lines, and shares 98.8% identity with a wheat EST sequence (GenBank accession: CV759357), we predicted that the fragment might be a wheat orthologue of the *A. thaliana PMR5* gene and provisionally designated the putative gene *TaPMR5*.

**Figure 7:**
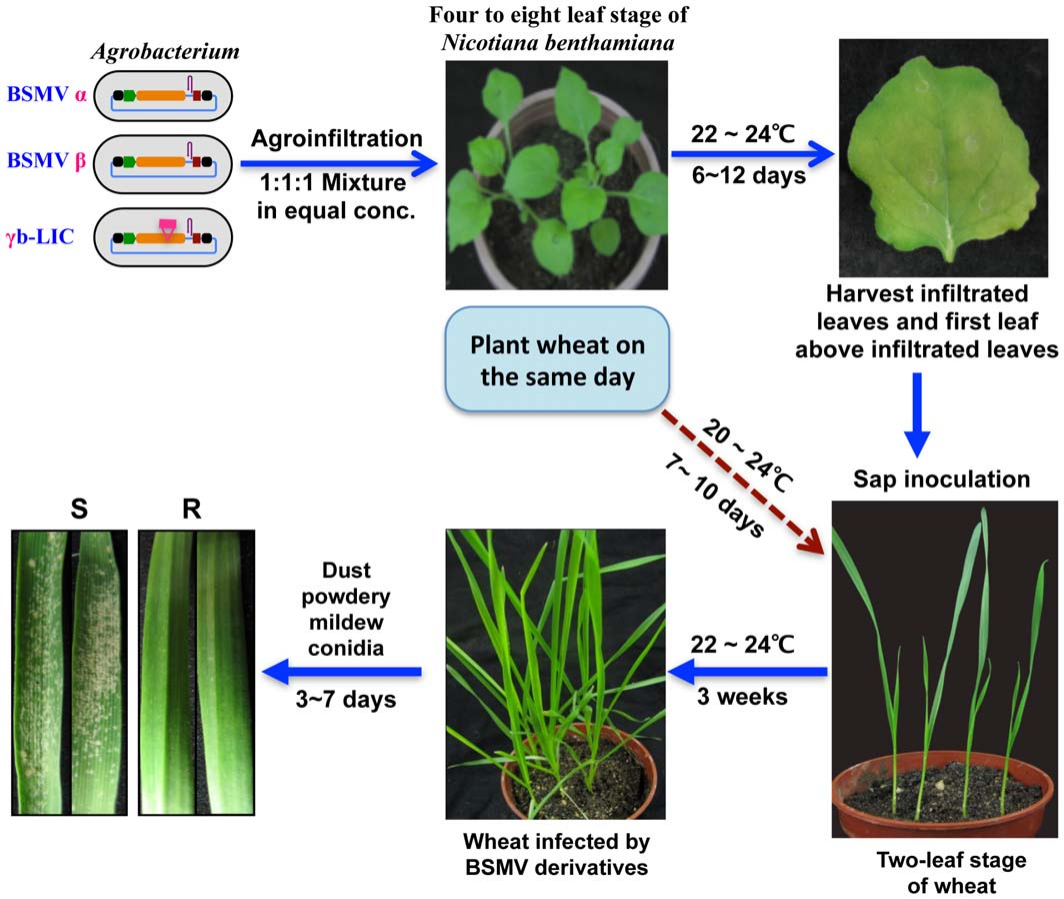
Flow chart showing the protocol used for analysis of the effects of *TaPMR5* silencing on powdery mildew invasion. The timing of *N. benthamiana* agroinfiltrations and use of infiltrated leaves for secondary inoculation of wheat in coordination with applications of powdery mildew conidia are illustrated.

To determine the effects of *TaPMR5* on wheat powdery mildew (*B. graminis* f. sp. *triticum*) infections, a 170 bp fragment was amplified from wheat DNA with the primer pair PMR5-8/PMR5-10 (Table S1) and inserted into pCa-γbLIC in the sense orientation to generate pCa-γb:*TaPMR5*_170_. This derivative was infiltrated into *N. benthamiana*, and sap from the infected leaves was inoculated to the first two emerging leaves of the highly susceptible wheat line Xuezao to assess the effects on mildew pathogenesis (Fig. 7). BSMV:00 (pCa-γbLIC) was tested as a vector alone control, and BSMV:*TaPDS*_200_ was used to confirm viral infection and spread.

**Figure 8:**
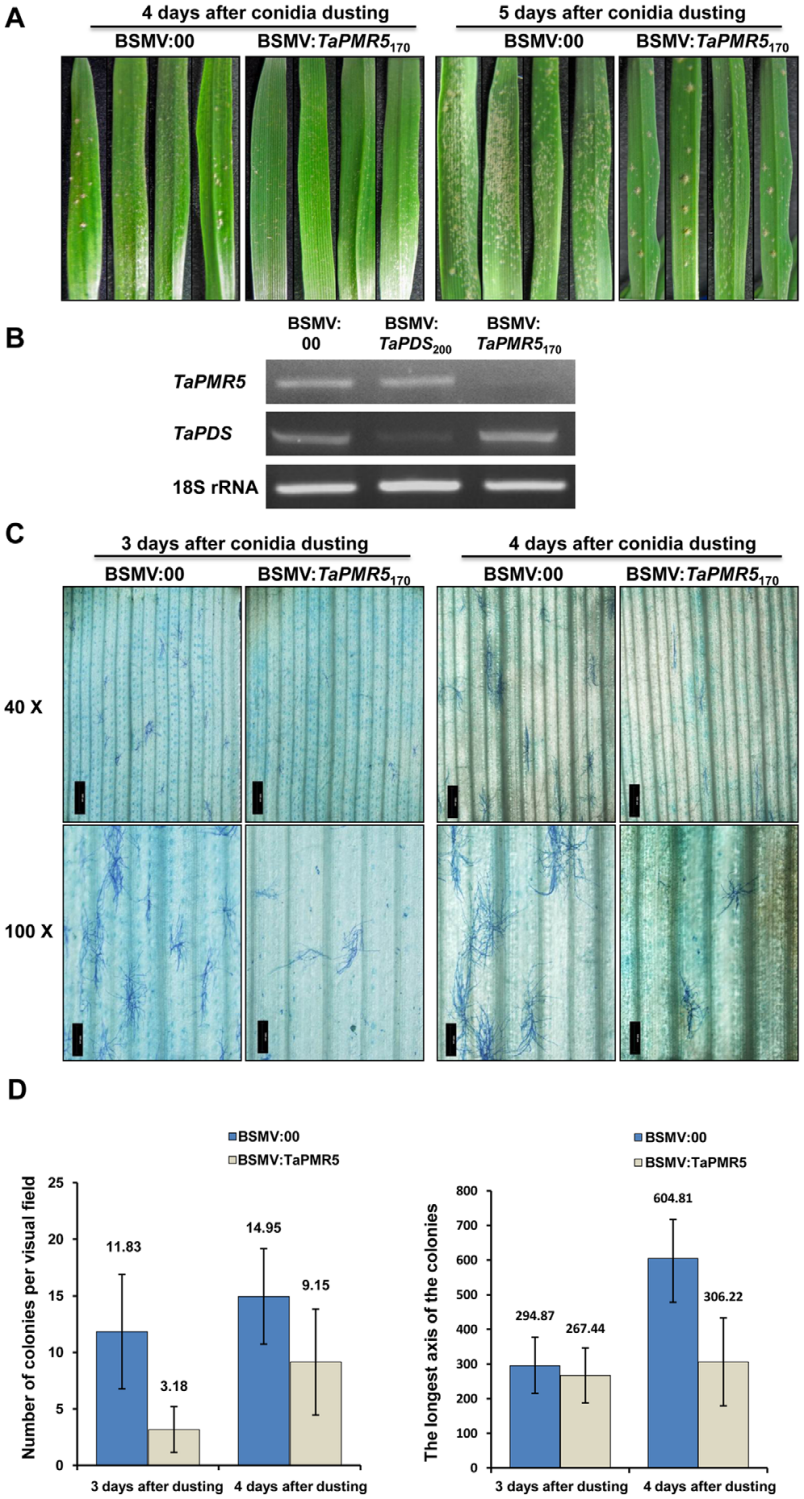
Effects of down regulation of *TaPMR5* on powdery mildew pathogenesis. (**A**) Highly susceptible wheat line (Xuezao) plants with visible symptoms of pCa-γbLIC (BSMV:00) or pCa-γb:*TaPMR5*_170_ (BSMV:*TaPMR5*_170_) challenged by dusting with *B. graminis* f. sp. *triticum* conidia. The photographs depict colonization of the 4th leaf emerging after inoculation with BSMV:00 or BSMV:*TaPMR5*_170_, and show the extent of mycelial development at 4 and 5 days after conidial dusting. (**B**) Relative transcript levels of *TaPMR5* and *TaPDS* in the 4th leaves emerging after infection with the BSMV derivatives (BSMV:00, BSMV:*TaPDS*_200_ or BSMV:*TaPMR5*_170_). RNA extracted from the leaves was subjected to semi-quantitative RT-PCR amplification (35 cycles) with the gene-specific oligonucleotide primers shown in Table S1. Amplified wheat 18S rRNA served as an internal control. (**C**) Higher resolution Olympus IX71 microscope observations of powdery mildew colonies appearing on leaf segments at 3 and 4 days after infection with BSMV:00 or BSMV:*TaPMR5*_170_. Leaf tissue was fixed in 75% ethanol/25% glacial acetic acid, and stained with 0.05% Coomassie blue R250. The bars represent 500 µm (40X) or 200 µm (100X). (**D**) Statistical analyses of colony formation and mycelia growth in wheat after *TaPMR5* suppression. The numbers of colonies per visual field were counted and the longest axis of the colonies was measured at 3 and 4 days after conidial applications. The ±SD analyses and error bars reflect the means of three independent experiments.

The potential effects of *TaPMR5* silencing on powdery mildew infections were assessed at 3 weeks after BSMV:00 or BSMV:*TaPMR5*_170_ inoculation of Xuezao plants. Colonies emerged on all plants by 4 days after dusting with conidia, but the number of colonies on BSMV:*TaPMR5*_170_ inoculated leaves were obviously lower than those on the BSMV:00 treated leaves at 4 and 5 days after mildew infection (Fig. 8A). By 11 days after conidia applications, most BSMV:00 treated plants had wilted and displayed extensive mildew colonization and growth, whereas the BSMV:*TaPMR5*_170_ treated plants were more robust and had much lower levels of mildew development (data not shown).

*TaPMR5* mRNA suppression in plants used for the mildew trials were evaluated by using the primer pair TaPMR5-7/TaPMR5-14 in RT-PCR reactions to amplify a 337 bp sequence from RNA of plants infected for 3 weeks with the three BSMV VIGS derivatives. As an internal control, the primer pair SQ18S-1/SQ18S-2 was used to amplify wheat 18S rRNA. The results show that leaves infected with BSMV:00, BSMV:*TaPDS*_200_ or BSMV:*TaPMR5*_170_ had similar levels of 18S rRNA transcripts (Fig. 8B). However, the *TaPMR5* transcript abundance was much lower in BSMV:*TaPMR5*_170_ infected plants than in the BSMV:00 or BSMV:*TaPDS*_200_ infections, and *TaPDS* RNA levels were specifically reduced in BSMV:*TaPDS*_200_ infected leaves (Fig. 8B). These results demonstrate that suppression of *TaPMR5* transcripts occurs only during infections with BSMV:*TaPMR5*_170_ sequences and provides an additional basis for the specificity of *TaPDS* VIGS.

For statistical evaluations of the mildew infections, the 4th emerging wheat leaves were cut into ∼4 cm long segments, placed in a Petri dish and dusted with powdery mildew conidia (Fig. 8C). The numbers of infection foci and hyphal growth between 3 and 4 days after conidial dusting revealed that leaves inoculated three weeks previously with BSMV:*TaPMR5*_170_ were significantly less susceptible to mildew infections than those infected with BSMV:00. In three sets of independent experiments in which more than 500 colonies were counted and measured for each treatment, the average numbers of colonies on leaf segments that had been infected with BSMV:00 and BSMV:*TaPMR5*_170_ respectively were 11.83±5.06 and 3.18±2.02 foci at 3 days after dusting with significant differences (P<0.0001), whereas colony numbers on leaf sections increased to 14.95±4.22 and 9.15±4.69 foci respectively at 4 days after dusting, with significant differences (P<0.0001) between the two groups (Fig. 8D). In addition, hyphal invasion rates were significantly lower in leaf segments infected with BSMV:*TaPMR5*_170_ than those infected with BSMV:00. The average sizes of the mildew colonies on BSMV:00 and BSMV:*TaPMR5*_170_-infected leaf segments were 294.87±82.16 µm and 267.44±79.32 µm respectively at 3 days after dusting with significant differences (P<0.01). The average colony sizes increased to 604.81±112.51 µm and 306.22±126.66 µm at 4 days after dusting, with significant (P<0.0001 differences) between the two groups (Fig. 8D). In sum, significant colony growth occurred between 3 and 4 days after application of conidia to leaf sections infected with BSMV:00, whereas foci in BSMV:*TaPMR5*_170_ infected leaves exhibited no significant differences in growth (Fig. 8C). Taken together, the data presented above indicate that down regulation of the *TaPMR5* gene has a substantial negative effect on rates of wheat powdery mildew infection, as was also the case with the previous *A. thaliana* powdery mildew infections [63].

## Discussion

BSMV VIGS vectors provide a reliable and efficient VIGS system and are now contributing to substantial advances in functional genomics research in a diverse range of monocot hosts [3]. The most frequent studies have focused on the natural host, barley, and on wheat, which is occasionally infected in the field. However, short communications have shown that BSMV VIGS can be used for down regulation of genes in *B. distachyon* [40], [41] and *Dasyprum villosum* (*H. villosa*) [38], [39], as well as the more distantly related monocot, culinary ginger (*Z. officinale*) [42]. In addition to these plants, selective BSMV strains can be mechanically transferred to a wide range of experimental hosts, including maize (*Zea mays*), rice (*Oryza sativa*), hexaploid oat (*Avena sativa*) and diploid oat (*A. strigosa*) [41], [69]. Hence, it is likely that BSMV VIGS can be applied more widely to other crop species. In this regard, we (Li Lab members; unpublished) have shown that BSMV ND18 infected barley sap can be used to establish systemic infections of millet (*Setaria italic*), several inbred maize lines and other *Triticum* species. Among these, wild emmer (*Triticum turgidum* var. *dicoccoides*, AABB, 2n = 4X = 28), the progenitor of cultivated tetraploid wheat, has particular promise as a donor of powdery mildew resistance genes [70]. Crosses of wild emmer with cultivated tetraploid durum wheat developed obvious photobleaching phenotypes on emerging leaves at 14 dpi when wheat *PDS* sequences (*TaPDS_200_*) were used for BSMV VIGS (data not shown) and this finding permits the powdery mildew resistance in these hybrids to be evaluated in more detail. In addition, use of the Agro/LIC silencing strategy should permit more effective and widespread applications of BSMV VIGS for high-throughput functional genomics of sorghum (*Sorghum bicolor*), *B. distachyon*, and other cereals, and to assist in evaluating gene functions before cross species introgression or transformation.

Among dicots, *N. benthamiana* is being developed rapidly for molecular genetic and genomics analyses, and our results show that BSMV VIGS provides another tool for functional genomics of this host. In addition to *N. benthamiana*, BSMV has been reported to elicit systemic infections in spinach (*Spinacia oleracea*) and a limited number of other dicots [69]. Primarily inoculated leaves of *Chenopodium* species, tobacco (*N. tabacum*) and beet (*Beta vulgaris*) also develop local infections [69] that might serve to initiate VIGS. These results suggest that the BSMV Agro/LIC strategy has potential for high-throughput functional genomics of susceptible dicots, as well as untested members of the Zingiberales and possibly other monocot families. Hence, BSMV VIGS appears to be well positioned to contribute to a more refined understanding of plant biology in the future [31].

Infections of barley and wheat with *in vitro* transcripts [25], [28] and bombardment with BSMV plasmids [26], [27] has been used with considerable success with VIGS studies of host genes involved in disease responses. In order to improve on these methods for large-scale functional genomics, we have developed agroinfiltration to permit secondary BSMV transfers from infiltrated *N. benthamiana* leaves and have introduced a LIC insertion site to facilitate efficient gene cloning. The silencing efficiencies of the resulting BSMV Agro/LIC VIGS vectors is comparable to those of the first generation systems [3], and the inserts appear to be very stable in infiltrated leaves. In addition to being a useful amplification host for BSMV, *N. benthamiana* can be grown under a wide range of temperature, humidity and light intensity conditions, and the 4 to 8 leaves that can be infiltrated on a single plant accumulate high levels of BSMV from 6 to 12 dpi under our growth conditions. In addition, infiltrated leaves or sap can be frozen or used to purify recombinant BSMV derivatives for subsequent experiments. Moreover, the inexpensive leaf sap inoculation procedure is very convenient and can be used to facilitate reliable and stable VIGS analyses of large numbers of plants. Taken together, our data indicate that the BSMV Agro/LIC VIGS strategy provides substantial advantages over previous BSMV VIGS systems in expense, cloning efficiency, ease of manipulation and ability to rapidly scale up preliminary experiments for high throughput genomics trials. To facilitate the use of the BSMV VIGS vectors, we will honor all requests for plasmids and other resources, and we encourage interested researchers to contact DL (Dawei.Li@cau.edu.cn) for their distribution. We also plan to deposit the vectors into a resource comparable to that of the Arabidopsis Biological Resource Center (ABRC) as soon as a comparable resource becomes available for cereal genomics.

Host susceptibility genes (S-genes) have been intensely studied in recent years to enable better understanding of pathogenesis and to provide alternative targets for durable and broad spectrum resistance breeding [71]. *A. thaliana PMR5* represents one such gene that has a role in infection processes of two powdery mildew fungi, *G. cichoracearum* and *G. cichoracearum* [63]. *PMR5* also affects rice blast (*Magnaporthe oryzae*) infections in in *A. thaliana* [72], but to date has not been reported to be involved in disease development of other fungal or bacterial pathogens. Other PMR genes identified in the *Arabidopsis* screen, such as *PMR4* and *PMR6* [63], [73], [74], also contribute to microbe-plant interactions in a resistance gene (R-gene) independent manner, and have been proposed to be S-genes [71], [75], [76]. The recessive *PMR5* gene encodes a novel protein that localizes in the endoplasmic reticulum and is predicted to participate in pectin biosynthesis, modification or deposition. This has lead to suggestions that PMR5 (and PMR6, which is a putative wall degrading enzyme) may alter wall composition sufficiently to interfere with critical host defense pathways or signaling and recognition requirements for hyphal penetration [77].

**Figure 9:**
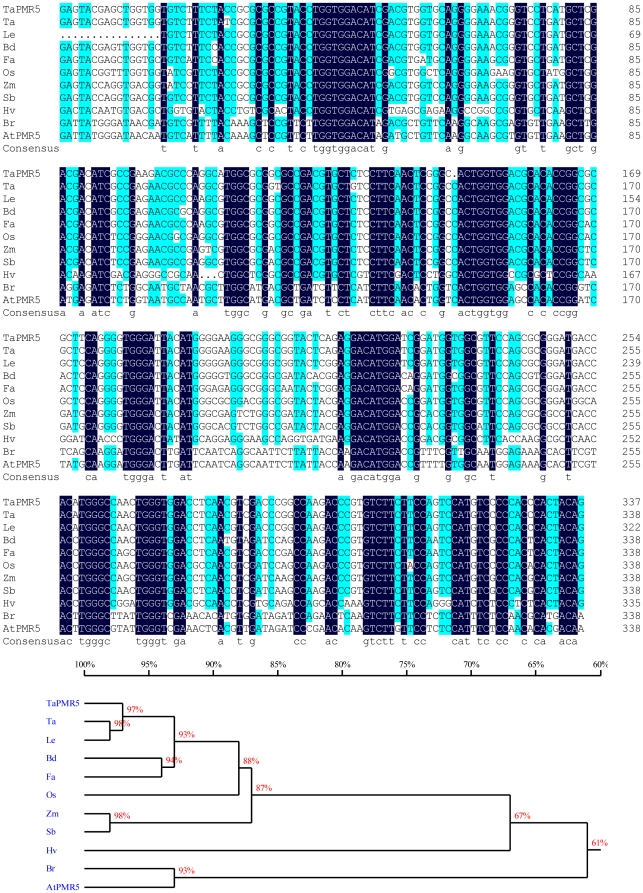
Multiple alignments (top) and phylogenetic analyses (bottom) of the 337 bp *TaPMR5* gene sequence with similar cDNA or EST sequences in wheat, barley, rice, maize, sorghum, *B. distachyon*, a species of fescue (*Festuca arundinacea*) and an interspecific hybrid of two wild rye species (*Leymus cinereus* x *Leymus triticoides*). This region has 90.3% identity in these cereal plants. *A. thaliana PMR5* gene and its Chinese cabbage orthologue were used as control outgroups. Nucleotides with white lettering in black boxes are 100% conserved, turquoise shading with white lettering indicates 75% or greater conservation, and unshaded nucleotides with black letters are less than 75% conserved. A phylogenetic tree of the PMR5 gene fragments from different species was obtained by analysis with DNAMAN software (Version 5.2.10, Lynnon corporation, Quebec, Canada). Ta  =  *Triticum aestivum* (GenBank accession: CV759357); Le  =  *Leymus cinereus* x *Leymus triticoides* (EG378784); Bd  =  *B. distachyon* (ADDN01000398); Fa  =  *Festuca arundinacea* (DT707549); Os  =  *Oryza sativa* (NM_001056332); Zm  =  Z*ea mays* (NM_001148816); Sb  =  *Sorghum bicolor* (XM_002467995); Hv  =  *H. vulgare* (BE413258); Br  =  *Brassica rapa* subsp. *Pekinensis* (EX125594); At * =  A. thaliana* (AK117382).

To identify potential PMR5 cereal orthologous, we conducted a BLAST search with the 337 bp *TaPMR5* sequence, which revealed the presence of related cDNA or EST sequences in wheat, barley, rice, maize, sorghum, *B. distachyon*, fescue species (*Festuca arundinacea*), and a wild rye interspecific hybrid (*Leymus cinereus* x *Leymus triticoides*). Multiple alignments of the 337 bp sequence revealed >90% sequence identity in these cereals when the *A. thaliana PMR5* gene and the Chinese cabbage orthologue provided a control outgroup (Fig. 9). These results support previous suggestions that *PMR5*, like other PMR genes involved in powdery mildew invasion [78], represents an ancient lineage whose functions have been conserved across the plant kingdom over ∼200 million years since divergence of the monocots and dicots [79].

Our results clearly show that down regulation of *TaPMR5* in a susceptible wheat line has a pronounced interference with the early stages of powdery mildew infection. Moreover, the resulting disease phenotype appears to mimic that previously noted with the *Atpmr5* mutant [63]. Both the *AtPMR5* allele and VIGS down regulation of *TaPMR5* result in reduced hyphal growth during the initial stages of infection and in reduced sporulation at later stages of powdery mildew infection, rather than blocking specific infection steps. Although additional work needs to be carried out to more thoroughly define *TaPMR5* regulatory interactions in cereal disease pathogenesis and roles in development, *Arabidopsis* studies have revealed that *AtPMR5* mutant cells are significantly smaller than wild type cells and chemical analyses showed elevated pectin and possibly aberrant pectin cross-linking [63]. Cell wall composition has become increasingly implicated in pathogen infection and defense responses, and the available evidence suggests that *PMR5* along with other PMR genes have diverse roles in formation of wall complexity elements needed for powdery mildew infections [77]. Although, the pectin composition of Type 1 cell walls of dicots differs substantially from Type 2 cell walls of the grasses [80], [81], our current results suggest that certain of these features have been conserved during evolution, and that BSMV Agro/LIC VIGS can provide a valuable tool for more refined understanding of the roles of these elements and the functions of “loss-of-susceptibility” orthologous during pathogenesis.

## Materials and Methods

### Plant material and growth conditions

*N. benthamiana* plants were grown in a controlled environmental climate chamber at 20–25°C with a 14/10 h light (∼75 µmol/m^2^.s)/dark photoperiod. Wheat (Yangmai 11), barley (Black Hulless) and *B. distachyon* (Bd21-3) plants used for VIGS experiments were grown in a greenhouse until the two leaf-stage, then inoculated with BSMV and transferred to a climate chamber at 23–25°C until evaluated. For powdery mildew experiments, the highly susceptible common wheat line Xuezao was inoculated with infected *N. benthamiana* sap and grown at 23–25°C under a 16/8 h light (∼160 µmol/m^2^.s)/dark regimen.

### Construction of BSMV VIGS vectors with LIC cloning sites

The pCass4-Rz [58] *Agrobacterium* plasmid was used for construction of VIGS vectors. Full-length α, β and γ cDNAs were amplified from BSMV ND18 clones [64] with high-fidelity *Pfu* DNA polymerase (Tiangen, Beijing, China) with the primer pairs BS-22/BS-32, BS-23/BS-26, or BS-24/BS-26, respectively (Table S1). The cDNAα clone was inserted into the *Stu*I site, and the cDNAβ and γ clones were integrated between the *Stu*I and *Bam*HI sites of pCass4-Rz, and the resulting clones were confirmed by sequencing, and designated pCaBS-α, pCaBS-β and pCaBS-γ (Fig. 1A).

A LIC site (5′-TAAGGAAGGGCCCGGTGGTGGTGGTTA-3′) was generated by inserting a 21 nt sequence (5′-GAAGGGCCCGGTGGTGGTGGT-3′) containing an *Apa*I restriction enzyme site (underlined sequence) into pCaBS-γ at the 3′ terminus of the γb gene to facilitate efficient cloning. The LIC vector was constructed by amplifying pCaBS-γ DNA with the primer pair BS-24/LIC-1 to produce fragment 1 containing cDNAγ residues 1–2536 fused to the 3 nt LIC sequence upstream of the *Apa*I site. A second amplification with the primer pair LIC-2/BS-32 generated fragment 2, whose sequence consisted of the *Apa*I site, the downstream 12 nt LIC sequence, and cDNAγ residues 2537–2790. Fragments 1 and 2 were then digested with *Apa*I or *Apa*I and *Bam*HI, mixed in equal amounts with *Stu*I and *Bam*HI-digested pCass4-Rz, and ligated to produce pCa-γbLIC (Fig. 1B). Similar strategies with different primer pairs (Table S1) were used to construct pCa-LICΔγb and pCa-LICγb (Fig. 1B).

The LIC strategy [59], was used to clone a series of *PDS*, *TK* [60] and magnesium chelatase subunit H (*ChlH*) [82] fragments. The selected sequences were amplified with primer pairs, in which the forward primers consisted of 5′-AAGGAAGTTTAA-3′ fusions to a 5′ plant sequence, and the reverse primers contained 5′-AACCACCACCACCGT-3′ fusions to each 3′ gene sequence. PCR products were purified, treated at room temperature with T4 DNA polymerase (New England Biolabs, MA, USA) in 1X reaction buffer containing 5 mM dATP for 30 min to generate sticky ends, and heat-treated at 75°C for 10 min to inactivate the polymerase. The *Apa*I-linearized pCa-γbLIC vector was also treated with T4 DNA polymerase in the presence of 5 mM dTTP to generate sticky ends complementary to those of the PCR products. The PCR products (∼200 ng) and pCa-γbLIC vector (20 ng) were mixed, incubated at 66°C for 2 min and slowly cooled to room temperature to anneal their complementary termini. Then, 10 µl aliquots were used for cloning into *Escherichia coli* DH10B, and transformants identified by colony PCR and endonuclease digestions.

### BSMV gene silencing derivatives for VIGS assessment

The *N. benthamiana* 370 bp *PDS* (*NbPDS*; GenBank accession: AJ571700) and 400 bp plastid transketolase (*NbTK*; GenBank accession: HQ200305) fragments were obtained by RT-PCR using the NbPDS-1/NbPDS-2 and NbTK-1/NbTk-2 primer pairs respectively (Table S1). The resulting fragments were integrated into pCa-γbLIC in the sense orientations to generate pCa-γb:*NbPDS*_370_ and pCa-γb:*NbTK*_400_. In a similar fashion, 200 and 400 bp wheat *PDS* (*TaPDS*; GenBank accession: FJ517553), 250, 300 and 547 bp wheat *ChlH* (*TaChlH*; TIGR accession: TC169257), 300 bp barley *ChlH* (*HvChlH*; GenBank accession: U26545), 300 and 400 bp barley *PDS* (*HvPDS*; GenBank accession: AY062039), and 102, 303 and 402 bp *B. distachyon PDS* (*BdPDS*; GenBank accession: HQ317869) fragments were amplified by RT-PCR with appropriate primer pairs (Table S1) and inserted into pCa-γbLIC.

### Agroinfiltration of *N. benthamiana* and viral inoculation of cereals

The BSMV pCa-γbLIC derivatives were transformed into *A. tumefaciens* strain EHA105. For agroinfiltration, single colonies were grown overnight at 28°C with constant shaking in 3 ml of LB containing rifampicin (25 µg/ml) and kanamycin (100 µg/ml). Then, 0.5 ml of the cultures were used to inoculate 50 ml LB containing the same antibiotics and grown at 28°C for 10 to 12 h. Bacterial cells were pelleted at 2200 *g* for 10 min, resuspended in infiltration buffer [10 mM MgCl_2_, 10 mM 2-(*N*-morpholino) ethanesulfonic acid (MES), pH 5.2, and 0.1 mM acetosyringone] to ∼0.7 OD_600_ and incubated at room temperature for at least 3 h. For agroinfiltration, equal amounts of bacteria harboring pCaBS-α, pCaBS-β and pCa-γbLIC (or its derivatives) were mixed, and infiltrated into four to eight *N. benthamiana* leaves immediately above the cotyledons with a 1-ml needleless syringe. After maintenance in a growth chamber for 5 to 12 days post infiltration (dpi), the infiltrated leaves were harvested, ground in 20 mM Na-phosphate buffer (pH7.2) containing 1% celite, and the sap was mechanically inoculated onto the two-leaf stages of wheat, barley and *B. distachyon*.

### RNA extraction and semi-quantitative RT-PCR analysis

Total RNA was extracted with TRIzol reagent as described by the manufacturer (Invitrogen, Carlsbad, CA, USA), treated with DNase, and first-strand cDNA synthesis carried out with M-MLV reverse transcriptase (Promega, Madison, WI, USA). Target gene expression levels were monitored by semi-quantitative RT-PCR using gene-specific primers that anneal outside the region targeted for silencing (Table S1). The wheat 18S rRNA gene (GenBank accession: AY049040), barley 18S rRNA gene (GenBank accession: AK251731), *B. distachyon* 18S rRNA (GenBank accession: DV483201), and tobacco 18S rRNA (GenBank accession: HQ384692) were used as individual internal RNA controls for appropriate assays.

### Powdery mildew inoculations

At 3 weeks after inoculation with *N. benthamiana* leaf sap, wheat developing visible BSMV symptoms (stripe mosaic on the 3th leaf with a mild mosaic on the 4th leaf) was challenged with *B. graminis* f. sp. *triticum* isolate E09, a prevalent pathotype in the Beijing area, by dusting test plants with conidia from infected wheat. Plants were maintained as described above until mildew observations were recorded. For detached leaf observations of VIGS effects, the 4th leaf exhibiting BSMV symptoms was cut into ∼4 cm segments, dusted with mildew conidia and incubated in a growth chamber for 5 days in Petri dishes containing 0.7% (w/v) agar and 40 ppm benzimidazole in distilled water.

### Staining and Microscopy of Powdery Mildew Infected Wheat

Leaves used for mildew observations were stained according to the Wise lab protocol [26], [27] with minor modifications. Leaves were fixed in 75% ethanol/25% glacial acetic acid overnight, and subsequently rinsed twice with deionized water. Then, tissue was immersed in Coomassie blue stain (0.05% Coomassie blue R250, 10% acetic acid, 50% methanol and 40% H_2_O) for 10–15 min, and examined immediately or stored in buffer (20% glycerol and 5% acetic acid) for up to 48 h before observation with an Olympus IX71 microscope.

### VIGS efficacy and data collection

To score susceptibility of VIGS treated wheat leaves, visible colonies were counted and their sizes determined by microscopy at 3 and 4 days after mildew inoculation. Colony numbers per visual field were calculated, the longest axis of the colonies was measured and the data were subjected to statistical analyses. Differences between the groups were evaluated by using the Student's *t*-test, where *P*<0.01 was considered statistically significant.

## Supporting Information

Figure S1 Comparison of BSMV symptoms with *PDS* and *ChlH* silencing phenotypes at 10 to 14 days after inoculation. (**A**) Barley leaves to illustrate uninoculated plants (Mock), with BSMV systemic mosaic on the upper emerging leaves of plants inoculated with BSMV:00, and the *PDS* and *ChlH* silencing phenotypes on leaves of plants inoculated with BSMV:*HvPDS*_300_ and BSMV:*HvChlH*_300_, respectively. Compare the faint yellow BSMV mosaic with the more expansive *PDS* white photobleaching phenotype and the intense yellowing phenotype elicited by *ChlH* silencing. (**B**) Comparison of systemically infected *Brachypodium distachyon* plants showing BSMV symptoms and the *PDS* silencing phenotype. Note that the upper leaves of the BSMV- infected plant have a mild chlorotic mosaic whereas the PDS silenced plant inoculated with BSMV:*BdPDS*_303_ exhibits intense white chlorotic streaks on the second, third and fourth leaves emerging above the inoculated leaves. (**C**) *B. distachyon* leaves from uninoculated plants, mild systemic mosaic symptoms and *PDS* phenotype on leaves of plants inoculated with BSMV:00 and BSMV:*BdPDS*_303_.
(TIF)

Table S1 Primers used in vector construction and molecular analyses.
(PDF)

Table S2 Infection characteristics and VIGS phenotype details in cereals inoculated with *N. benthamiana* sap from infected leaves infiltrated with BSMV Agro/LIC VIGS derivatives.
(PDF)
