# Supplementary figures and images for: A High Throughput Barley Stripe Mosaic Virus Vector for Virus Induced Gene Silencing in Monocots and Dicots
Source: PLoS One. 2011 Oct 21;6(10):e26468. doi: 10.1371/journal.pone.0026468 (PMC3198768; doi:10.1371/journal.pone.0026468)

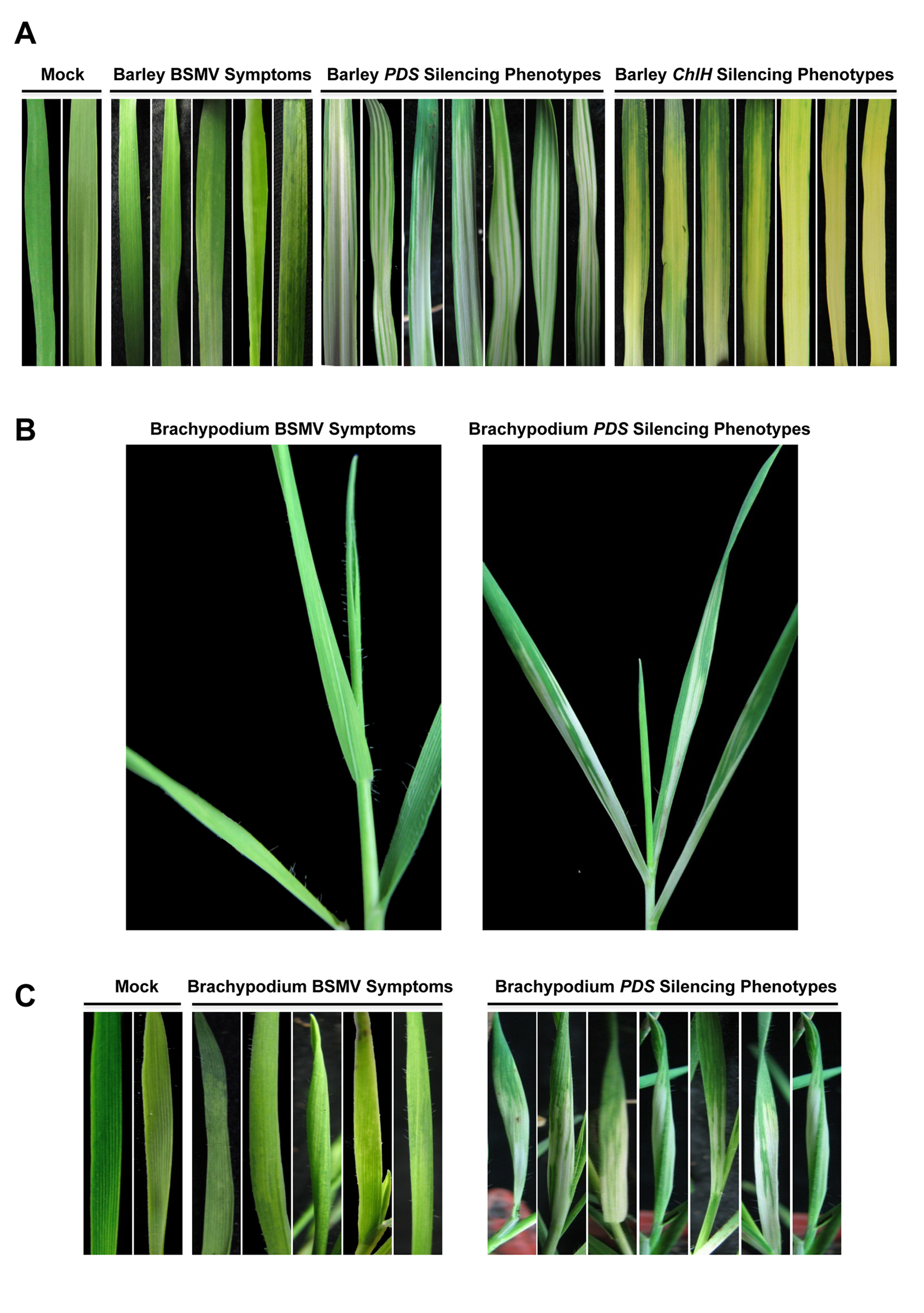

Supplement: Figure S1 — Comparison of BSMV symptoms with PDS and ChlH silencing phenotypes at 10 to 14 days after inoculation. (A) Barley leaves to illustrate uninoculated plants (Mock), with BSMV systemic mosaic on the upper emerging leaves of plants inoculated with BSMV:00, and the PDS and ChlH silencing phenotypes on leaves of plants inoculated with BSMV:HvPDS 300 and BSMV:HvChlH 300, respectively. Compare the faint yellow BSMV mosaic with the more expansive PDS white photobleaching phenotype and the intense yellowing phenotype elicited by ChlH silencing. (B) Comparison of systemically infected Brachypodium distachyon plants showing BSMV symptoms and the PDS silencing phenotype. Note that the upper leaves of the BSMV- infected plant have a mild chlorotic mosaic whereas the PDS silenced plant inoculated with BSMV:BdPDS 303 exhibits intense white chlorotic streaks on the second, third and fourth leaves emerging above the inoculated leaves. (C) B. distachyon leaves from uninoculated plants, mild systemic mosaic symptoms and PDS phenotype on leaves of plants inoculated with BSMV:00 and BSMV:BdPDS 303. (TIF) [file pone.0026468.s001.tif]
