# Supplementary material for: A High Throughput Barley Stripe Mosaic Virus Vector for Virus Induced Gene Silencing in Monocots and Dicots
Source: PLoS One. 2011 Oct 21;6(10):e26468. doi: 10.1371/journal.pone.0026468 (PMC3198768; doi:10.1371/journal.pone.0026468)
Supplement: Table S1 — Primers used in vector construction and molecular analyses. (PDF) [file pone.0026468.s002.pdf]

Table S1. Primers used in vector construction and molecular analyses

Primer	Primer sequence (5′–3′) [a]	Position and description [b]	Usage
BS-10	GGTGCTTGATGCTTTGGATAAGG	BSMV RNAγ nt 1864-1886	Verification of the stability of inserted fragments in pCa-γbLIC vectors in PCR with BS-32
BS-22	GTATGTAAGTTGCCTTTGGGTG	BSMV RNAα nt 1-22	pCaBS-α construction with BS-32
BS-23	GTAAAAGAAAAGGAACAACCCTG	BSMV RNAβ nt 1-23	pCaBS-β construction with BS-26
BS-24	GTATAGCTTGAGCATTACCGTC	BSMV RNAγ nt 1-22	pCaBS-γ construction with BS-26
BS-26	CGGGATCCTGGTCTTCCCTTGGGGGAC	*Bam*HI, BSMV RNAγ nt 2790-2772 (contains 19 nt common to the 3' termini of BSMV gRNAs)	Construction of pCaBS-β with BS-23, and pCaBS-γ with BS-24
BS-32	TGGTCTTCCCTTGGGGGAC	BSMV RNAγ nt 2790-2772 (contains 19 nt common to the 3' termini of BSMV gRNAs)	pCaBS-α construction with BS-22, and verification of the stability of inserted sequence fragments with BS-10
LIC-1	CCGGGCCCttcCTTAGAAACGGAAGAATCATCAC	*Apa*I, partial LIC sequence, BSMV RNAγ nt 2536-2514	pCa-γbLIC construction with LIC-2
LIC-2	CCGGGCCCggtggtggtggtTAAAAAAAAAAAAAAATG TTTGATCAG	*Apa*I, partial LIC sequence, BSMV RNAγ nt 2537-2563	Construction of pCa-γbLIC with LIC-1, and pCa-LICΔγb with LIC-3
LIC-3	CCGGGCCCttccttaGCGAAGGTAAATACAGTAG	*Apa*I, partial LIC sequence, BSMV RNAγ nt 2080-2062	Construction of pCa-LICΔγb with LIC-2, and pCa-LICγb with LIC-4
LIC-4	CCGGGCCCggtggtggtggtCTACTTTCTCTTGTGTGTG	*Apa*I, partial LIC sequence, BSMV RNAγ nt 2088-2106	pCa-LICγb construction with LIC-3
NbPDS-1	AAGGAAGTTTAACTGACGAGCTTTCGATG	LIC adaptor, *NbPDS* nt 21-36	pCa-γb:*NbPDS*$_{370}$ construction
NbPDS-2	AACCACCACCACCGTCATATATGGACATTTATC	LIC adaptor, *NbPDS* nt 390-373	
NbTK-1	AAGGAAGTTTAAACTACCTGCTGGCTG	LIC adaptor, *NbTK* nt 1-15	pCa-γb:*NbTK*$_{400}$ construction
NbTK-2	AACCACCACCACCGTTCCTAGACCAATTGAATC	LIC adaptor, *NbTK* nt 400-383	
SQNbPDS-1	TCCTCACGCCCAACTAAACCATT	*NbPDS* (GenBank accession: EU165355) nt 533-555	Semi-quantitative RT-PCR of *NbPDS* gene
SQNbPDS-2	CTTCAACATAAGATTGCCCTCCA	*NbPDS* nt 989-967	
SQNbTK-1	TGTCTGAGGCTGGAGTTATTTATG	*NbTK* gene nt 352-375	Semi-quantitative RT-PCR of *NbTK* gene
SQNbTK-2	CTTGTAGTCGGCTGATTGTTC	*NbTK* gene nt 794-774	
SQNb18S-1	GATCAGATACCGTCCTAGTC	Tobacco 18S rRNA nt 981-1000	Semi-quantitative RT-PCR of *N. benthamiana* 18S rRNA gene
SQNb18S-2	CCCGGAACCCAAAAACTTTG	Tobacco 18S rRNA nt 1093-1074	
TaPDS-1	AAGGAAGTTTAATTTCTCCAGGAGAAG	LIC adaptor, *TaPDS* nt 1029-1043	Pairing with TaPDS-2 or TaPDS-3

TaPDS-2	AACCACCACCACCGTCTGCATAAACGCTTAAAAG	LIC adaptor, *TaPDS* nt 1428-1410	pCa-γb:*TaPDS*$_{400}$ construction with TaPDS-1
TaPDS-3	AACCACCACCACCGTTCTCCAGTTATTTGAG	LIC adaptor, *TaPDS* nt 1228-1213	pCa-γb:*TaPDS*$_{200}$ construction with TaPDS-1
TaChl-1	AAGGAAGTTTAACGTCACCACCA	LIC adaptor, *TaChlH* nt 555-565	Pairing with TaChl-2, or TaChl-3, or TaChl-4
TaChl-2	AACCACCACCACCGTTCCTCCGGGTTACCGGACCC	LIC adaptor, *TaChlH* nt 854-835	pCa-γb:*TaChlH*$_{300}$ construction with TaChl-1
TaChl-3	AACCACCACCACCGTGGAGATGCCCTCCCTCTCCAC	LIC adaptor, *TaChlH* nt 804-784	pCa-γb:*TaChlH*$_{250}$ construction with TaChl-1
TaChl-4	AACCACCACCACCGTACACACCTGGGATATC	LIC adaptor, *TaChlH* nt 1101-1086	pCa-γb:*TaChlH*$_{547}$ construction with TaChl-1
HvChl-1	AAGGAAGTTTAAGACGAGGTGAACCTGCTCGACGACCATCTG	LIC adaptor, *HvChlH* nt 391-420	pCa-γb:*HvChlH*$_{300}$ construction
HvChl-2	AACCACCACCACCGTATCTTGCTCCTCCAAGTAGG	LIC adaptor, *HvChlH* nt 690-671	
HvPDS-1	AAGGAAGTTTAAAAGCCAGGAGAATACAGC	LIC adaptor, *HvPDS* nt 200-217	Pairing with HvPDS-2 or HvPDS-3
HvPDS-2	AACCACCACCACCGTACAGAATGCACTGCATAG	LIC adaptor, *HvPDS* nt 499-482	pCa-γb:*HvPDS*$_{300}$ construction with HvPDS-1
HvPDS-3	AACCACCACCACCGTAATGGTTAACAATAGGC	LIC adaptor, *HvPDS* nt 599-583	pCa-γb:*HvPDS*$_{400}$ construction with HvPDS-1
BdPDS-1	AAGGAAGTTTAAAAGCCAGGAGAATACAGC	LIC adaptor, *BdPDS* nt 49-66	Pairing with BdPDS-2, or BdPDS-3, or BdPDS-4
BdPDS-2	AACCACCACCACCGTCGGCCAAGTAAGCATTTC	LIC adaptor, *BdPDS* nt 150-133	pCa-γb:*BdPDS*$_{102}$ construction with BdPDS-1
BdPDS-3	AACCACCACCACCGTCAGAATGCACTGCATAG	LIC adaptor, *BdPDS* nt 351-335	pCa-γb:*BdPDS*$_{303}$ construction with BdPDS-1
BdPDS-4	AACCACCACCACCGTATGGTTAACAATAGGC	LIC adaptor, *BdPDS* nt 450-435	pCa-γb:*BdPDS*$_{402}$ construction with BdPDS-1
SQTaPDS-1	TTTCTCCAGGAGAAGCATGGCT	*TaPDS* nt 1029-1050	Semi-quantitative RT-PCR of *TaPDS* gene
SQTaPDS-2	GGACCTCGGTGTCTTCACAA	*TaPDS* nt 1640-1621	
SQTaChl-1	ACGACCATCTGGTGGATGTTCTGC	*TaChl* nt 731-754	Semi-quantitative RT-PCR of *TaChlH* gene
SQTaChl-2	TGCCTCAAACAGTTGGGAATCACG	*TaChl* nt 1235-1212	
SQHvPDS-1	TTTCTCCAGGAGAAGCATGGCT	*HvPDS* nt 518-539	Semi-quantitative RT-PCR of *HvPDS* gene
SQHvPDS-2	GGACCTCGGTGTCTTCACAA	*HvPDS* nt 1129-1110	
SQHvChl-1	ACGACCATCTGGTGGATGTTCTGC	*HvChlH* nt 410-433	Semi-quantitative RT-PCR of *HvChlH* gene
SQHvChl-2	CGGAGCCGATGCCTCAAAACAGTGGG	*HvChlH* nt 926-901	
SQBdPDS-1	GTAATCCTCCTGAAAGGCTATGC	*BdPDS* nt 410-432	Semi-quantitative RT-PCR of *BdPDS* gene
SQBdPDS-2	AGTTTGAAGATGTCAACTGGTGC	*BdPDS* nt 605-583	
SQ18S-1	GTGACGGGTGACGGAGAATT	Wheat 18S rRNA nt 349-368,<br>Barley 18S rRNA nt 1555-1574,<br>putative *B. distachyon* 18S rRNA nt 348-367	Semi-quantitative RT-PCR of wheat, barley and *B. distachyon* 18S rRNA gene
SQ18S-2	GACACTAATGCGCCCGGTAT	Wheat 18S rRNA nt 498-479,<br>Barley 18S rRNA nt 1705-1686,<br>putative *B. distachyon* 18S rRNA nt 498-479	

PMR5-7	GAGTACGAGCTGGTGGTGTCTTTCTACCGC	*TaPMR5* nt 1-30	Amplification of *TaPMR5* 377 bp and 170 bp gene fragments
PMR5-8	AAGGAAGTTTAAGAGTACGAGCTGGTGGTGTCTTTCTACCGC	LIC adaptor, *TaPMR5* nt 1-30	pCa-γb:*TaPMR5*$_{170}$ construction
PMR5-10	AACCACCACCACCGTCGCGCCGGTGTGCGTCCACCAGTGCCCGAG	LIC adaptor, *TaPMR5* nt 170-141	
PMR5-14	CTGTAGTGGGTGGGGGACATGGACTGGAAG	*TaPMR5* nt 337-308	Amplification of 377 bp *TaPMR5* gene fragment, and semi-quantitative RT-PCR of *TaPMR5* gene

[a] Underlined letters indicate restriction enzyme sites, lower case letters in primers LIC-1 to LIC-4 show partial LIC sequences, shadowed letters indicate adaptor sequences for target sequence amplification.

[b] Numbers correspond to target nucleotide positions; a reverse order of numbers indicates that the primer is complementary to the targeted sequences.
