# Supplementary material for: A High Throughput Barley Stripe Mosaic Virus Vector for Virus Induced Gene Silencing in Monocots and Dicots
Source: PLoS One. 2011 Oct 21;6(10):e26468. doi: 10.1371/journal.pone.0026468 (PMC3198768; doi:10.1371/journal.pone.0026468)
Supplement: Table S2 — Infection characteristics and VIGS phenotype details in cereals inoculated with N. benthamiana sap from infected leaves infiltrated with BSMV Agro/LIC VIGS derivatives. (PDF) [file pone.0026468.s003.pdf]

**Table S2**. Infection characteristics and VIGS phenotype details in cereals inoculated with *N. benthamiana* sap from infected leaves infiltrated with BSMV Agro/LIC VIGS derivatives.

<table>
<tr><th rowspan="2">Hosts</th><th rowspan="2">Varieties (line)</th><th rowspan="2">Target genes</th><th colspan="2">Infection Details [a]</th><th colspan="4">VIGS Results at 23-25°C [b]</th></tr>
<tr><th>Infection efficiency (%)</th><th>Symptom appearance (dpi)</th><th>Initial VIGS (dpi)</th><th>Optimal intensity (dpi)</th><th>Phenotype Appearance (dpi)</th><th>% of Infected Plants with VIGS</th></tr>
<tr><td rowspan="3">Wheat</td><td>Yangmai 11</td><td rowspan="3">TaPDS<br>TaChlH</td><td>80-90</td><td>5-6</td><td>10-11</td><td>12-14</td><td rowspan="3">> 30</td><td>> 80</td></tr>
<tr><td>Chinese Spring, Xiaoyan 54</td><td>80-90</td><td>7-9</td><td>10-11</td><td>10-14</td><td>> 80</td></tr>
<tr><td>Xuezao</td><td>50-60</td><td>7-9</td><td>11-12</td><td>13-15</td><td>> 70</td></tr>
<tr><td rowspan="2">Barley</td><td rowspan="2">Black Hulless, Yangfu 6032</td><td>BvPDS</td><td>80-90</td><td>5-6</td><td>10-11</td><td>12-14</td><td>> 30</td><td>> 80</td></tr>
<tr><td>BvChlH</td><td>80-90</td><td>5-6</td><td>10-11</td><td>11-15</td><td>>30</td><td>>80</td></tr>
<tr><td>B. distachyon</td><td>Bd21-3</td><td>BdPDS</td><td>80-90</td><td>6-7</td><td>11-12</td><td>11-12</td><td>>30</td><td>> 80</td></tr>
</table>

[a] Inoculum consisted of primary infiltrated *N. benthamiana* leaf sap for inoculation of the first two emerging cereals leaves. Plants were maintained at 23-25°C, under ~75 μmol/m$^2$.s light intensity with a 14/10 h light/dark regimen.

[b] Observations of silencing phenotype and evaluation of gene functions were performed on the 4th cereal leaf.
